# Rates and Determinants of Repeated Participation in a Web-Based	Behavior Change Program for Healthy Body Weight and Healthy Lifestyle

**DOI:** 10.2196/jmir.9.1.e1

**Published:** 2007-01-22

**Authors:** Marieke W Verheijden, Marielle P Jans, Vincent H Hildebrandt, Marijke Hopman-Rock

**Affiliations:** ^2^Body@WorkResearch Centre Physical ActivityWork and HealthTNO-VU University Medical CentreAmsterdamThe Netherlands; ^1^TNO Quality of LifeLeidenThe Netherlands

**Keywords:** Internet, counseling, health behavior change, selective enrollment, selective retention

## Abstract

**Background:**

In recent years, many tailored lifestyle counseling programs have become available through the Internet. Previous research into such programs has shown selective enrollment of relatively healthy people. However, because of the known dose-response relationship between the intensity and frequency of counseling and the behavior change outcomes, selective retention may also be a concern.

**Objective:**

The aim of this study was to identify rates and determinants of repeat participation in a Web-based health behavior change program.

**Methods:**

A Web-based health behavior change program aimed to increase people’s awareness of their own lifestyle, to promote physical activity, and to prevent overweight and obesity was available on the Internet from July 2004 onward at no cost. Univariate and multivariate logistic regression analyses were conducted to identify characteristics of people who participated in the program more than once. Age, compliance with physical activity guidelines, body mass index, smoking status, and the consumption of fruit, vegetables, and alcohol were included in the analyses.

**Results:**

A total of 9774 people participated in the baseline test, of which 940 used the site more than once (9.6%). After exclusion of individuals with incomplete data, 6272 persons were included in the analyses. Of these 6272 people, 5560 completed only the baseline test and 712 also participated in follow-up. Logistic regression predicting repeated use determined that older individuals were more likely to participate in follow-up than people aged 15-20 years. The odds ratios for the age categories 41-50, 51-60, and > 60 years were 1.40 (95% CI = 1.02-1.91), 1.43 (95% CI = 1.02-2.01), and 1.68 (95% CI = 1.03-2.72), respectively. Individuals who never smoked were more likely to participate repeatedly than current smokers and ex-smokers (OR = 1.44, 95% CI = 1.14-1.82 and OR = 1.49, 95% CI = 1.17-1.89, respectively). People meeting the guidelines for physical activity of moderate intensity (OR = 1.23 95% CI = 1.04-1.46) and for vegetable consumption (OR = 1.26 95% CI = 1.01-1.57) were also more likely to participate repeatedly than people who did not, as were obese people compared to individuals with normal weight (OR = 1.41 95% CI = 1.09-1.82).

**Conclusions:**

For some variables, this study confirms our concern that behavioral intervention programs may reach those who need them the least. However, contrary to most expectations, we found that obese people were more likely to participate in follow-up than people of normal body weight.  The non-stigmatizing way of addressing body weight through the Internet may be part of the explanation for this. Our findings suggest that Web-based health behavior change programs may be more successful in the area of weight management than in many other health-related areas. They also stress the importance of adequate coverage of weight management in Web-based health promotion programs, as a driver to continue participation for overweight and obese people.

## Introduction

In recent years, many tailored health promotion programs have become available through the Internet. As with any other health-promoting intervention, these Web-based health promotion programs are not expected to lead to either short-term or sustained behavior change unless the intervention reaches the intended target audience. Unfortunately, it has been postulated that health-promoting lifestyle interventions tend to reach those who need them the least [1]. For ethical and practical reasons, there is often no information available on people who decide not to participate (see, for example, the study by Sirard and colleagues [2]). Dutta-Bergman [3] showed that people who look for health information on the Internet are more health oriented than people who don’t look for such information. They are also more likely to hold stronger health-oriented beliefs and to engage in healthy activities. Similarly, Verheijden et al [4] conducted a nonresponse survey and showed that participants in a Web-based tailored nutrition counseling program were a relatively well-educated and healthy subsample of the target audience. It is thus evident that efforts need to be made to minimize selective enrollment in Web-based health promotion programs.

Furthermore, it is known that there is a strong dose-response relationship between the number and intensity of counseling sessions and behavior change outcomes [5-7]. It is therefore unlikely that a difficult process such as health behavior change can be achieved in a single counseling session; repeated participation is likely necessary to achieve sustainable changes. It seems reasonable to assume that this is also true for Web-based counseling, which means that repeated exposure to the counseling programs is necessary to achieve sustainable changes.

In addition to selective enrollment in Web-based programs, selective attrition during follow-up may thus be a concern too. This concern was also expressed by Eysenbach [8] and by Danaher and colleagues [9], who argued that attrition, uptake, exposure, and diffusion measures need to be addressed in addition to the effectiveness of eHealth programs. Indeed, little is known about attrition and its determinants in Web-based behavior change programs. The current study therefore addresses the rates and health and lifestyle determinants of repeated participation in a Web-based health behavior change program. The current focus on user characteristics is in line with the relevance of these characteristics that was made explicit by Christenson and Mackinnon [10].

## Methods

### The Web-Based Health Promotion Program

The Web-based health promotion program on which the current paper is based was a Web-based version of the Dutch National Health Test, designed by the Netherlands Organisation for Applied Scientific Research TNO in cooperation with the Dutch Foundation Pur Sang. The program was developed with funding from the Dutch Ministry of Health, Welfare and Sport and aimed to increase people’s awareness of their own lifestyle, to promote physical activity, and to prevent overweight and obesity. The program was available on the Internet from July 2004 onward at no cost. The launch of the site was brought to the public’s attention with a press release presenting the State Secretary as the very first participant in the program. No further media and marketing strategies were used to keep the program in the public’s view. However, various organizations were interested in the program, which generated free publicity. For example, articles on the Dutch National Health Test were published in national and local newspapers, in free magazines published by several supermarket chains, in women’s magazines, and on websites of general practices and municipal health services.

**Figure 1:**
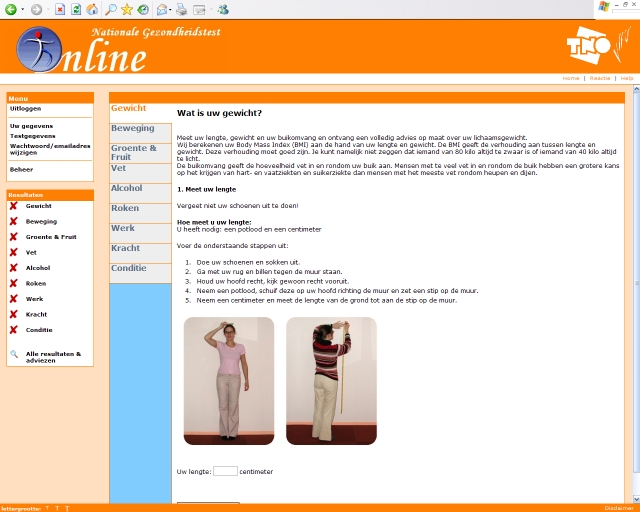
Screenshot of the Dutch National Health Test (Nationale Gezondheidstest Online)

The registration procedure for the website included selection of a personal username and password and a series of questions on sociodemographic characteristics of the participants. The health promotion program contained modules on anthropometrics (height, weight, waist circumference), physical activity, dietary habits, alcohol intake, smoking, work, cardiorespiratory fitness, and muscle strength. The modules on lifestyle consisted of a series of questions to assess the current behavior. The modules on anthropometrics, cardiorespiratory fitness, and muscle strength contained instructions for the appropriate self-tests. Upon completion of each individual module, participants received feedback that was tailored to the responses they had given in that module. Because the physical activity module was the core of the program, additional questions on physical activity were included. Figure 1 presents a screenshot of the Dutch National Health Test (in Dutch). Additional screenshots are available in the Multimedia Appendix (in Dutch). The Dutch National Health Test is no longer available to the public. More information can be obtained from the authors.

The tailored feedback messages for the individual modules were also integrated in an overall report. Participants could print this report and keep it for their own reference. The data were stored in a database and used as a basis for longitudinal feedback in follow-up participation. The data were also kept for research purposes. Participants’ consent for this was obtained. Participants were allowed to complete the modules over a two-week period. They were sent an email reminder to complete the modules in a timely manner. During the initial participation, people were made aware of the availability of follow-up modules. They were encouraged to participate in these follow-up modules to monitor their progress and to receive more tailored feedback. Invitations for follow-up participation were sent out by email three months after the completion date of the last entry. The program was made available online in June 2004, which means that the first reminders for follow-up could have been sent out in October 2004; however, due to a technical error in the automated reminders, the first reminders were not sent out until March 2005.

### The Independent Variable: Participation

Although each individual module led to immediate tailored feedback, people were not considered to be participants unless they had completed the modules on anthropometrics and physical activity. Single participation was therefore defined as having only one record in the Dutch National Health Test database in which the modules on anthropometrics and physical activity were completed. Repeat participation was defined as having two or more records in the Dutch National Health Test in which the modules on anthropometrics and physical activity were completed.

### Predictors for Repeat Participation

Data on gender, age (15-20, 21-30, 31-40, 41-50, 51-60, 61 and older), and education level (very low, low, intermediate, high, very high) were obtained in the registration process of the Web-based health promotion program. The body mass index was calculated using data on self-reported height and weight. It was subsequently categorized (≤ 25 kg/m^2^, 25.01-30 kg/m^2^, > 30 kg/m^2^). People were encouraged to measure and report their waist circumference (in centimeters). Detailed instructions on measuring waist circumference, which included some clear pictures, were provided. Smoking status was defined as currently smoking, formerly smoking, or never having smoked. Physical activity was categorized based on the criteria for sufficient physical activity of (1) moderate intensity (moderate intense physical activity at least 5 days per week for at least 30 minutes per day) and (2) high intensity (intense physical activity at least 3 days per week for at least 20 consecutive minutes). Current Dutch guidelines define minimum intake levels for sufficient fruit and vegetables. For fruit, the guideline is a minimum of two pieces per day; for vegetables, the guideline is a minimum of 200 g per day. Participants were categorized as either meeting or not meeting the guidelines for fruit and vegetable consumption. Alcohol consumption was defined based on current Dutch gender-specific guidelines, which define the maximum number of alcoholic drinks per week (ie, a maximum of 15 alcoholic drinks per week for women and 21 for men). People exceeding these numbers were defined as excessive drinkers.

### Analysis

Descriptive statistics were used to present data on the baseline characteristics of the participants of the Web-based health promotion program. No data on waist circumference will be presented because inspection of the data revealed that people likely gave invalid answers. For example, the reported waist circumferences varied from as little as 20 cm to as much as 2 km. Furthermore, values such as 50 cm, 60 cm, 70 cm, and 80 cm were reported much more frequently than values such as 53 cm, 69, and 77 cm. Descriptive statistics were also used to present data on single and repeated use of the Web-based health promotion programs. These analyses are based on data from the 9774 people who met the participation requirements of this program (ie, people who completed questions on anthropometrics and physical activity).

Univariate and multivariate logistic regression analyses were conducted to identify the crude and adjusted effects of factors associated with repeat participation in a Web-based health behavior change program. A total of 9774 people enrolled in the Web-based health promotion program, but because of missing data, all regression analyses were based on 6272 participants. The exclusion of 3502 people was the result of missing values in the variables on smoking (missing for 3010 people) and on the consumption of fruit (missing for 2751 people), vegetables (missing for 2751 people), and alcohol (missing for 3399 people). Analyses comparing the characteristics of people with and without missing values revealed no clinically relevant differences. People with missing values, for example, were significantly older than people without missing values (*P* < 0.01), but the difference was less than one year.

## Results

### Participants and Number of Visits to the Web-Based Health Promotion Program

Approximately two thirds of the 9774 participants were female. The mean age was 36 years (SD = 13). The vast majority of the participants (90.1%) had an intermediate or (very) high education level. The mean body mass index was 24.5 kg/m^2^ (SD = 5.7). At the time of enrollment, 22% of the participants were smokers. The guidelines for physical activity of moderate intensity and high intensity were met by 51% and 46% of participants, respectively. Few people met the guidelines for fruit and vegetable consumption (22% and 14%, respectively), and 7% consumed more alcoholic drinks per week than recommended by current Dutch gender-specific guidelines.

Of the 9774 people who enrolled in the Web-based health promotion program, almost 10% participated more than once: 7.6% participated twice, and 1.9% participated three times. The completion of four visits was very infrequent (< 1%).

### Determinants of Repeat Participation

People aged 41 years and older repeatedly participated in the intervention more than those aged 15-20 years (Table 1). In the univariate analyses, a healthy lifestyle was related to repeat participation. For example, repeat participation was more frequent among former smokers (OR = 1.73) and people who never smoked (OR = 1.47) than among current smokers. It was also more frequent among people with sufficient physical activity than among people with insufficient physical activity (OR = 1.31 for moderate intensity activity, OR = 1.23 for high intensity activity). Finally, repeated participation was more frequent among people meeting the guidelines for fruit consumption (OR = 1.26) and vegetable consumption (OR = 1.39) than among those failing to meet the guidelines. In contrast to the pattern that a healthy lifestyle was related to repeat participation, people who were overweight (OR = 1.20) or obese (OR = 1.54) more frequently participated repeatedly than people of normal body weight.

In the multivariate analyses, repeat participation was more frequent among people aged 41 years and older (OR = 1.40-1.68), among obese people (OR = 1.41), among former smokers and individuals who never smoked (OR = 1.49 and 1.44, respectively), among people with insufficient physical activity of moderate intensity (OR = 1.23), and among people with a sufficient vegetable consumption (OR = 1.26) than among the relevant reference groups.

**Table 1:** Participants’ baseline characteristics as determinants of repeat participation in a Web-based health behavior change program (bold numbers indicate significant OR values), N = 6272

	**Repeat Participation (%)**		**Crude Effect**		Adjusted Effect^*^	
			**OR**	**95% CI**	**OR**	**95% CI**
**Gender**						
Male (n = 2132)	11.7		1.00	–	1.00	–
Female (n = 4140)	11.2		0.95	0.80-1.11	0.99	0.83-1.18
**Age (years)**						
15-20 (n = 846)	9.3		1.00	–	1.00	–
21-30 (n = 1717)	8.6		0.92	0.69-1.22	0.97	0.72-1.32
31-40 (n = 1449)	12.0		1.33	1.00-1.75	1.31	0.97-1.79
41-50 (n = 1281)	13.1		**1.47**	**1.11-1.95**	**1.40**	**1.02-1.91**
51-60 (n = 791)	14.2		**1.60**	**1.18-2.18**	**1.43**	**1.02-2.01**
61 and older (n = 188)	16.6		**1.92**	**1.23-3.03**	**1.68**	**1.03-2.72**
**Education level**						
Very low (n = 159)	12.6		1.00	–	1.00	–
Low (n = 421)	12.6		1.00	0.58-1.74	0.90	0.51-1.58
Intermediate (n = 1838)	11.8		0.93	0.57-1.52	0.88	0.53-1.46
High (n = 1082)	10.8		0.84	0.51-1.40	0.83	0.49-1.39
Very high (n = 2272)	11.0		0.86	0.53-1.39	0.77	0.46-1.27
**Body mass index (kg/m^2^)**						
≤ 25 (n = 4044)	10.4		1.00	–	1.00	–
25.01-30 (n = 1662)	12.3		**1.20**	**1.01-1.44**	1.11	0.92-1.33
> 30 (n = 566)	15.2		**1.54**	**1.20-1.88**	**1.41**	**1.09-1.82**
**Smoking status**						
Current smoker (n = 1343)	8.1		1.00	–	1.00	–
Former smoker (n = 2092)	13.2		**1.73**	**1.37-2.18**	**1.49**	**1.17-1.89**
Never smoker (n = 2837)	11.5		**1.47**	**1.17-1.84**	**1.44**	**1.14-1.82**
**Physical activity, moderate intensity^†^**						
Insufficient (n = 3009)		9.9	1.00	–	1.00	–-
Sufficient (n = 3263)		12.7	**1.31**	**1.12-1.54**	**1.23**	**1.04-1.46**
**Physical activity, high intensity^‡^**						
Insufficient (n = 3377)		10.4	1.00	–	1.00	–
Sufficient (n = 2895)		12.5	**1.23**	**1.05-1.44**	1.11	0.94-1.31
**Vegetable consumption^§^**						
Insufficient (n = 5409)		10.9	1.00	–	1.00	–
Sufficient (n = 863)		14.5	**1.39**	**1.13-1.71**	**1.26**	**1.01-1.57**
**Fruit consumption^||^**						
Insufficient (n = 4919)		10.8	1.00	–	1.00	–
Sufficient (n = 1353)		13.2	**1.26**	**1.05-1.50**	1.06	0.87-1.28
**Alcohol consumption^¶^**						
Excessive (n = 420)		11.2	1.00	–	1.00	–
Moderate or none (n = 5852)		11.4	0.98	0.72-1.35	1.02	0.74-1.41

^*^The regression model contained repeat participation (yes/no) as the dependent variable and gender, age, education level, body mass index, smoking status, physical activity (moderate and high intensity), fruit consumption, vegetable consumption, and alcohol consumption as independent variables.

^†^Current guidelines in The Netherlands recommend a total of at least 30 minutes of physical activity of moderate intensity at least 5 days per week.

^‡^Current guidelines in The Netherlands recommend at least 20 consecutive minutes of physical activity of high intensity at least 3 days per week.

^§^Current guidelines in The Netherlands recommend at least 200 g of vegetables per day.

^||^Current guidelines in The Netherlands recommend at least 2 pieces of fruit per day.

^¶^Current guidelines in The Netherlands recommend a maximum of 15 alcoholic drinks per week for women and 21 drinks per week for men.

## Discussion

This study showed that people who repeatedly participated in a Web-based health promotion program generally had healthier lifestyles than people who participated only once. In contrast to this and to our expectations, people who were overweight or obese participated more frequently than people of normal body weight. Repeated use was relatively infrequent; approximately 10% of the people used the program more than once.

The initial concern in reaching the appropriate target audience with Web-based health promotion programs is to prevent selective enrollment in the program. An extensive comparison of the baseline characteristics of the participants in the current study with the Dutch population in general is beyond the scope of this article. A birds-eye view of the baseline characteristics, however, indicates that the participants in the Web-based program had a lower prevalence of overweight and obesity and smoking, and a higher prevalence of compliance with the guidelines for physical activity and fruit and vegetable consumption than the Dutch population in general.

As was discussed recently by Eysenbach [8], eHealth applications face the difficulty that a (sometimes substantial) proportion of people will not be using the application or will be using it sparingly. The latter was also true for the Dutch National Health Test. Our study confirms our hypothesis that selective retention in Web-based health behavior change programs is a concern in addition to selective enrollment. The group of participants who used the program repeatedly were a relatively healthy subsample of all people who enrolled in the program. The only exception to this was for body weight, as people who were overweight or obese used the program more frequently than people of normal body weight. One explanation for this is that a higher risk for disease is associated with a higher probability of participating in counseling [11]. It is known that overweight and obese people perceiving weight as a health risk are more likely to have prepared and/or initiated activities to lose weight [12]. Furthermore, people with chronic conditions are more likely to search for health information on the Internet than those without [13]. Another possible explanation for the fact that overweight and obese people used the program relatively frequently is that Web-based counseling may be particularly appealing for people with stigmatizing diseases. Despite the increasing prevalence of overweight and obesity in most groups of the population [14,15], excessive body weight or the failure to lose weight may continue to be stigmatizing [16-18]. This may also help explain the unexpected effects that were observed for body weight in the current study.

Given the known dose-response relationship between the frequency and intensity of counseling and the achieved behavior change outcomes, it is disappointing that only 10% of the participants used the program more than once. Previous research on Web-based health behavior change programs has shown that people are much less interested in programs that encourage lifestyle improvement than they are in programs that simply compare their behavior to relevant guidelines [19]. This comparison to relevant guidelines can be achieved with a single participation. On the other hand, when people are looking for solid counseling on possible lifestyle improvements, multiple counseling sessions are necessary. People’s lack of interest in behavior change counseling may thus help to explain the limited repeated use of the current program.

It is unclear how people’s motivation to participate in behavior change counseling may be increased, but it is evident that this change needs to be brought about before Web-based health promotion programs have the potential to lead to sustained behavior change. Upon first use of the program, an effort should be made to explain that behavior change does not occur overnight and that the program people are working with includes follow-up modules that make long-term support possible. Work presented by Spittaels and De Bourdeaudhuij [20] suggests some other approaches that may contribute to increased use of Web-based behavior change programs. A key issue may be to have face-to-face contact before people are referred to the Web-based program. When 100 flyers with information on a Web-based program to promote physical activity were handed out to participants in person, it led to 41 people receiving tailored advice. When the same number of flyers were placed in strategic positions throughout a hospital, it led to only 8 people receiving tailored advice. Another factor that was emphasized in the work of Spittaels and De Bourdeaudhuij [20] is the use of frequent reminder emails. These reminder emails were appreciated by the study participants, but no effect in terms of self-reported physical activity was observed. Rewarding people with something may also help to increase repeated participation [19]. Verheijden and colleagues reported that 76% of the people who were not intrinsically motivated to participate in follow-up programs said they would be interested in participating when given a bonus or reward.

In conclusion, our findings support the debate on the current proliferation of Web-based health behavior change programs and they stress the need to find new approaches to reach the primary target groups via the Web. This study supports earlier findings that Web-based health behavior change programs may largely fail to reach those for whom health behavior change is most necessary. By interesting contrast, overweight and obese people were more frequently repeat users than people of normal body weight. This effect may be due in part to the non-stigmatizing nature of Web-based interventions as opposed to face-to-face interventions. These findings suggest that Web-based health behavior change programs may be more successful in the area of weight management than in many other health-related areas. It also stresses the importance of adequate coverage of weight management in Web-based health promotion programs, as a driver to continue participation for overweight and obese people.

## Multimedia Appendix

Screenshots of the Dutch National Health Test 
      	
