# Supplementary figures and images for: Rates and Determinants of Repeated Participation in a Web-Based Behavior Change Program for Healthy Body Weight and Healthy Lifestyle
Source: J Med Internet Res. 2007 Jan 22;9(1):e1. doi: 10.2196/jmir.9.1.e1 (PMC1794672; doi:10.2196/jmir.9.1.e1)

## Slide 1
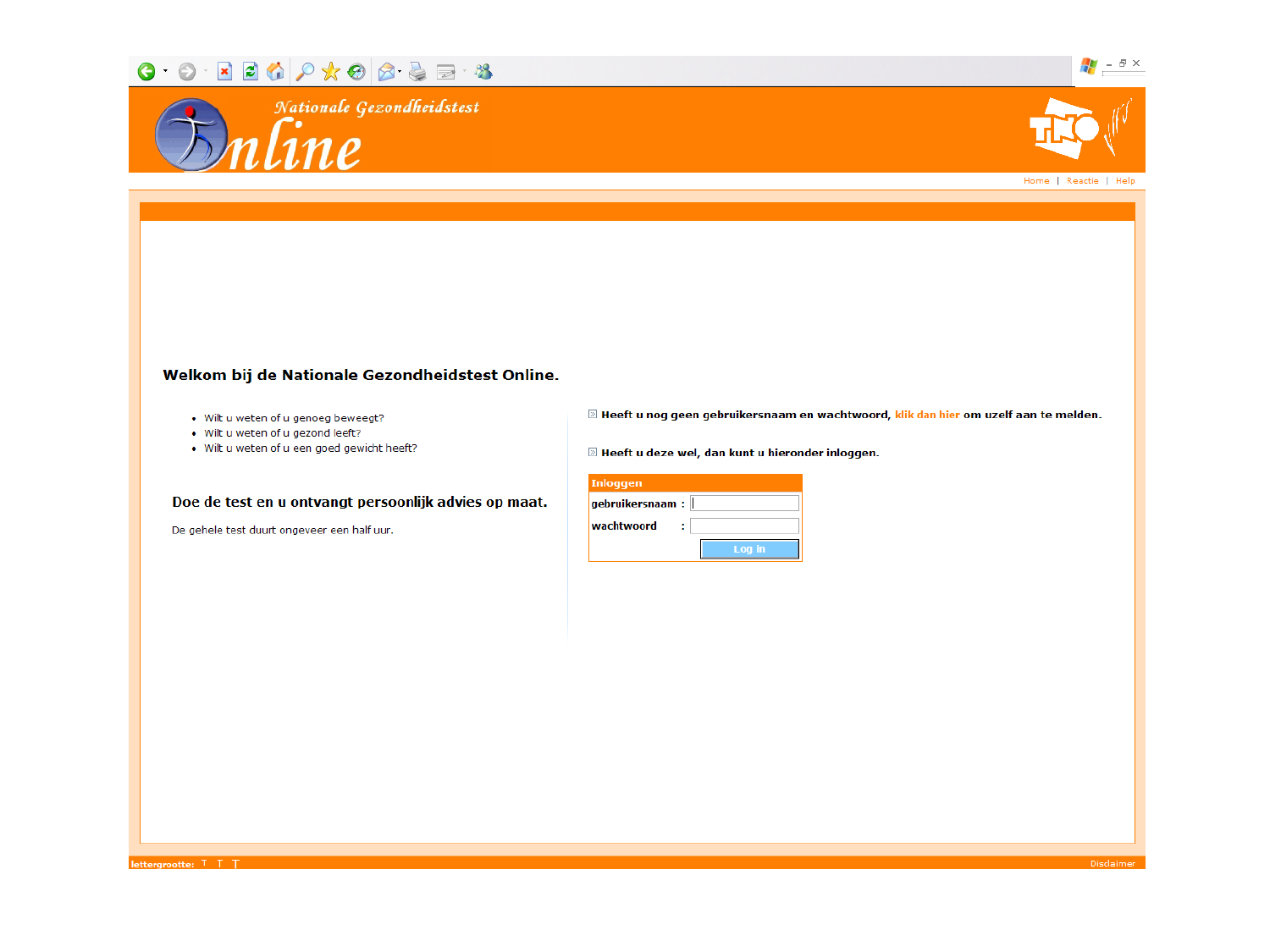

## Slide 2
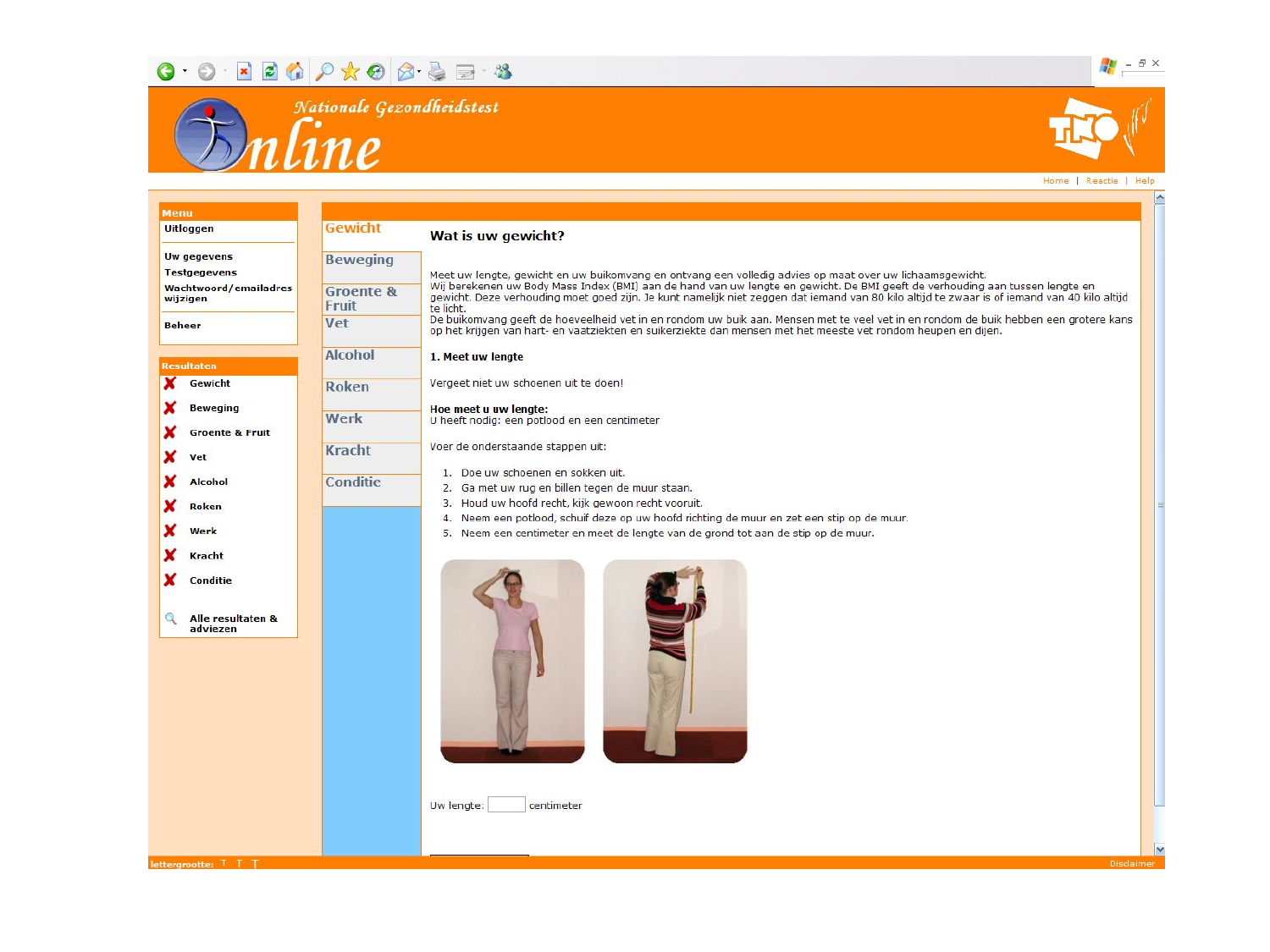

## Slide 3
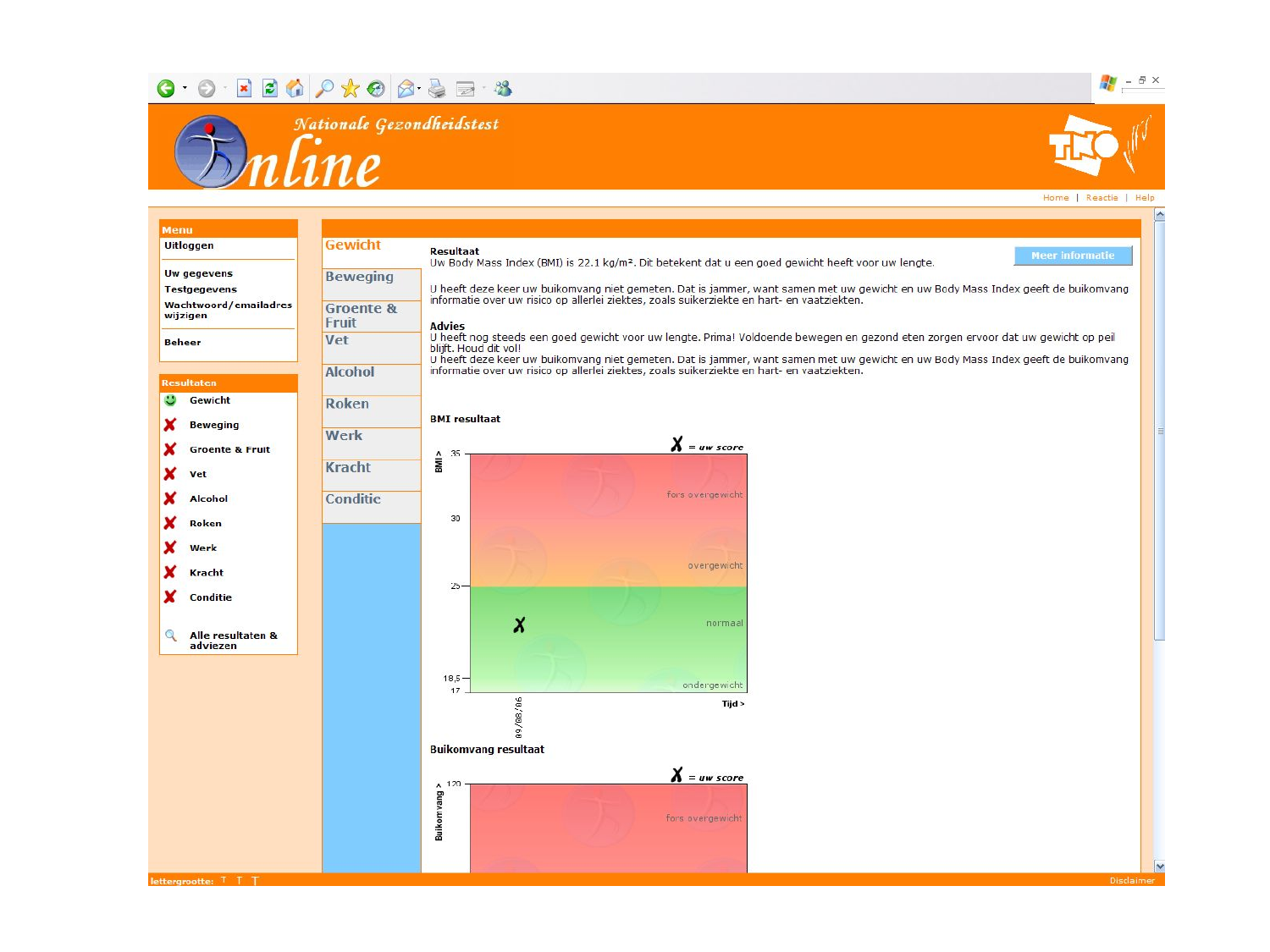

## Slide 4
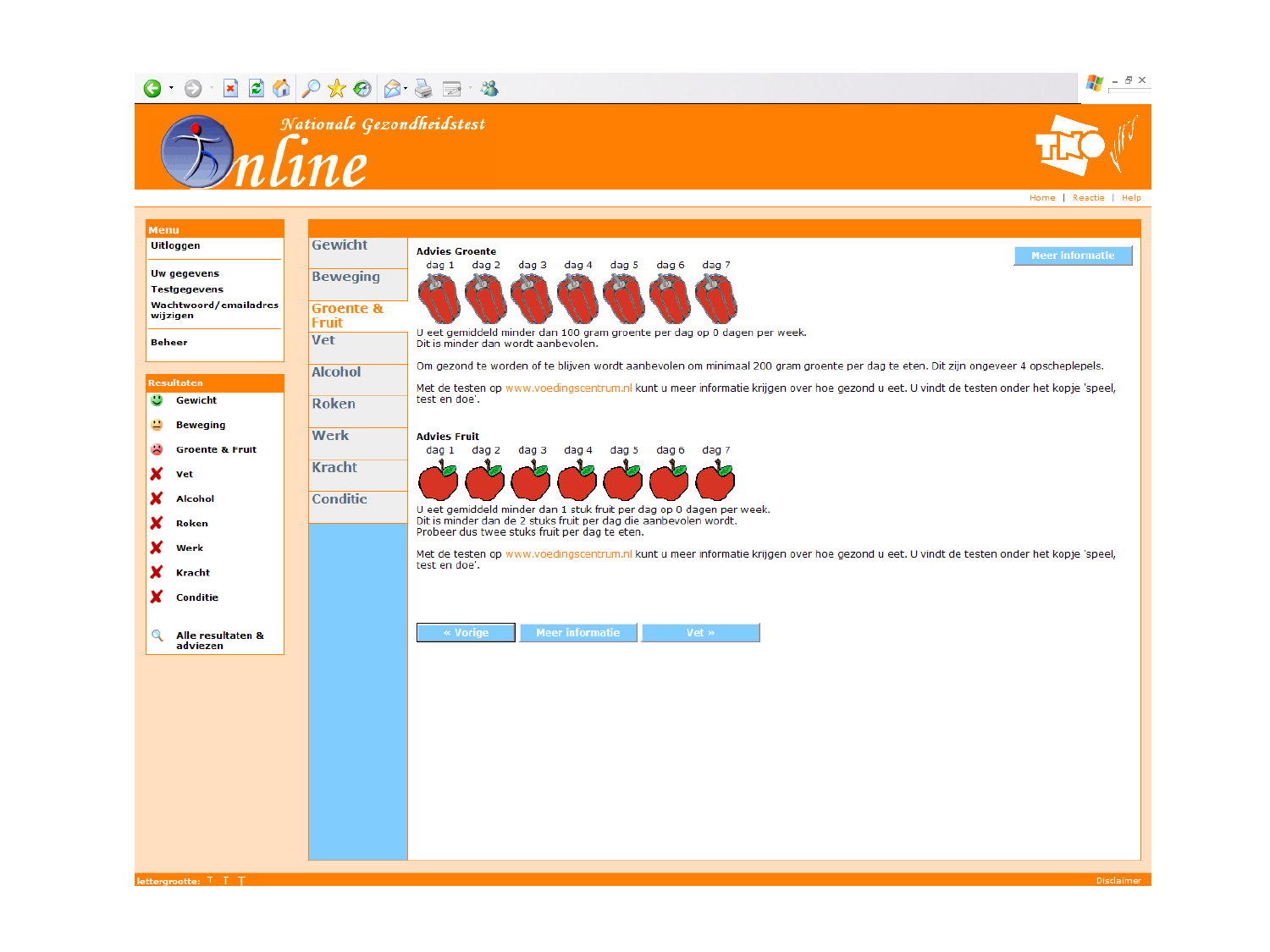

## Slide 5
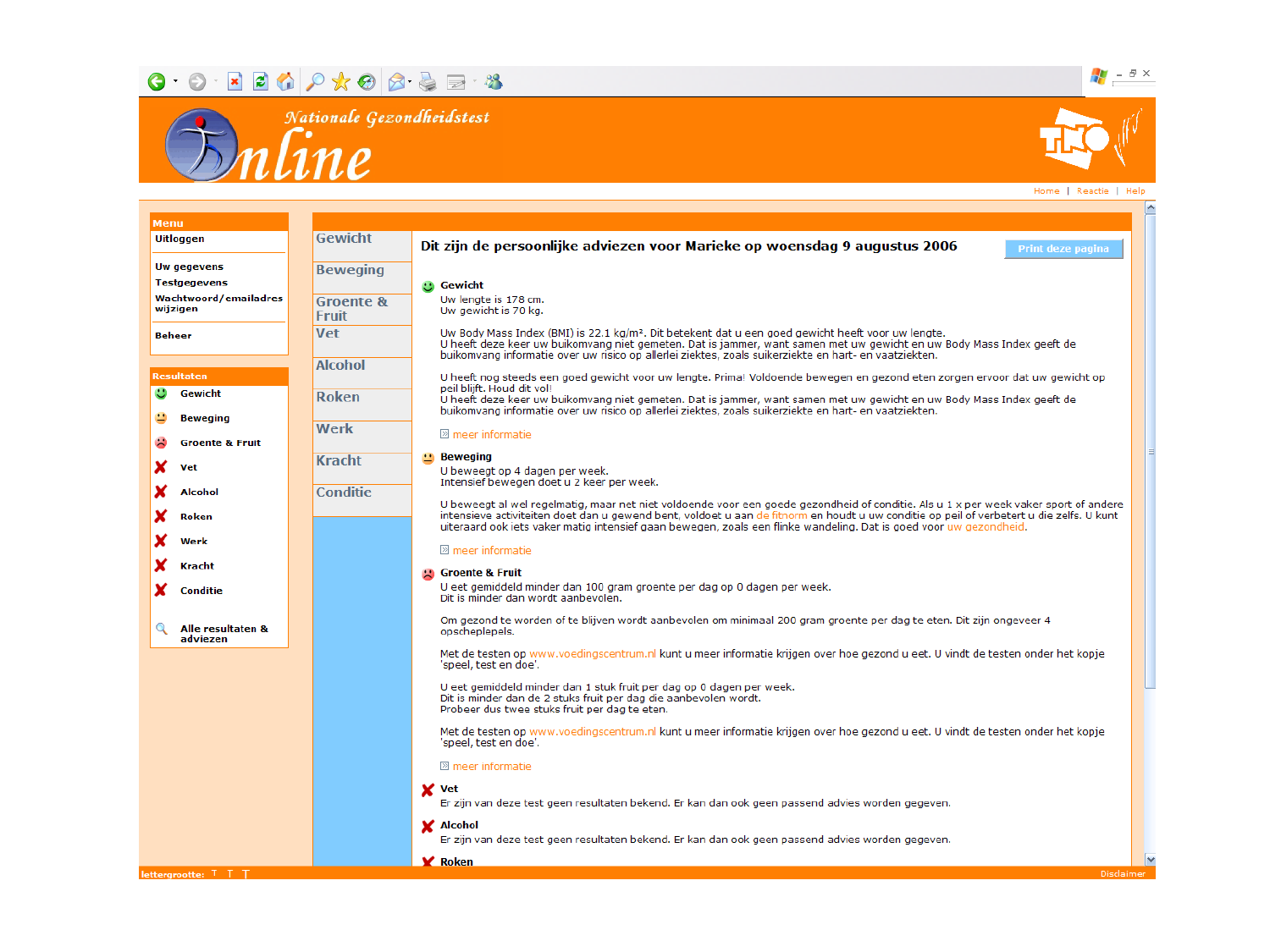

Supplement: Supplementary file 1 [file jmir_v9i1e1_app1.ppt]
